# DNAM-1: would the real natural killer cell please stand up!

**DOI:** 10.18632/oncotarget.5952

**Published:** 2015-09-28

**Authors:** Nicholas D. Huntington, Ludovic Martinet, Mark J. Smyth

**Affiliations:** Immunology in Cancer and Infection Laboratory, QIMR Berghofer Medical Research Institute, Herston and School of Medicine, University of Queensland, Herston, Queensland, Australia

**Keywords:** Immunology and Microbiology Section, Immune response, Immunity, NK cells, maturation, cytokine, DNAM-1, effector

Natural Killer (NK) cells were called as such due to their ability to spontaneously kill tumor cell lines *in vitro*. Peripheral NK cells have long been considered rapid responding innate effector lymphocyte primed to produce cytotoxic granules and pro-inflammatory cytokines such as IFN-γ and TNF-α when they encounter virusinfected, transformed or damaged self-tissue. However, it is becoming clear that considerable heterogeneity exists with the peripheral NK cell pool and researchers should reconsider the generalized view of NK cell effector function and contribution to mammalian immunity. This is extremely relevant to tissues such as the liver, salivary gland and intestine where type 1 innate lymphoid cells (ILC1) are found and resemble NK cells in many regards yet have distinct transcription factor requirements to conventional NK cells.

For over 10 years now we have known that NK cells of various maturational stages can be found in tissues such as the spleen, lymph nodes, liver and lung and these NK cells exhibit differential responsiveness to homeostatic cytokines and effector responses. These differential responses were attributed to the alternate maturation state however a recent report from Martinet et al. has revealed that functional heterogeneity exists amongst NK cells of similar maturation based on DNAM-1 expression [1]. DNAM-1 (CD226) is a cell surface glycoprotein that functions as an adhesion molecule to synergize with activating receptors and trigger NK cellmediated cytotoxicity upon interaction with its ligands CD155 and CD112 [2]. The authors found that DNAM-1 expression is unanimous on NK cell progenitors, however is down-regulated as NK cells mature, generating DNAM-1^+^ and DNAM-1− NK cells in the periphery. The ratio of DNAM-1^+^ NK cells to DNAM-1− NK cells diminishes from birth and the DNAM-1− subset does not appear to revert to a DNAM-1^+^ state. DNAM-1 expression did not correlate with other known markers of NK cell maturation such as KLRG1 [3] and CD27/CD11b [4] with both KLRG1^+^ and CD27/CD11b NK cell subsets expressing similar proportions of DNAM-1^+^ and DNAM-1− NK cells. Instead, DNAM-1 expression was strongly linked to NK cell effector functions with DNAM-1^+^ NK cells producing significantly more IFN-γ, IL-6, CCL5, and GM-CSF, but less MIP1α/β than their DNAM-1− counterparts following stimulation with IL-12 and IL-18 [1]. DNAM-1^+^ NK cells also contributed greater IFN-γ secretion than DNAM-1− NK cells *in vivo* following TLR agonist treatment and suppressed tumor growth and metastasis *in vivo* more effectively, even independently of DNAM-1 function. DNAM-1^+^ NK cells were transcriptionally distinct and were extremely sensitive to IL-15 compared with their DNAM-1− counterparts. Given that up-regulation of IL-15 by accessory cells is a response to pathogen detection, this suggests that the DNAM-1^+^ NK cells are the likely subset primed in such infections.

**Figure 1:**
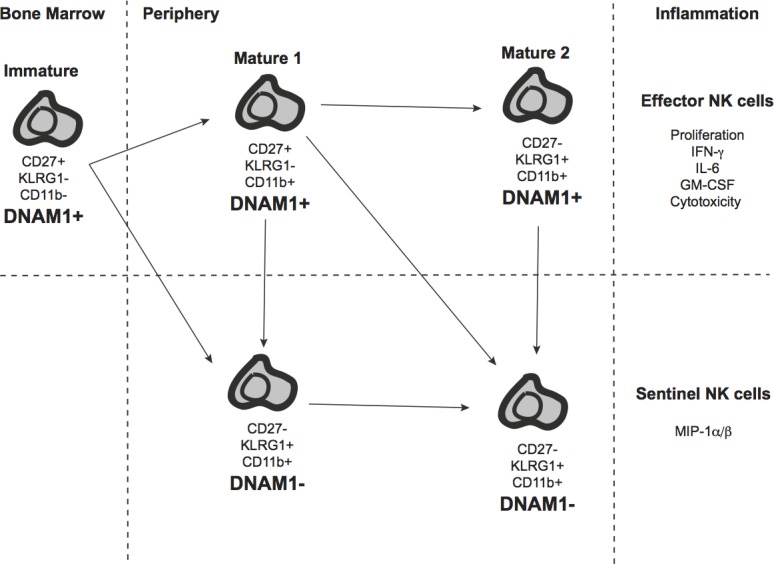
The DNAM-1 axis of function DNAM-1^+^ Immature NK cells develop in the bone marrow from NK cell progenitors and migrate and differentiate into Mature 1 DNAM-1^+^ NK cells in peripheral lymphoid organs. At this stage, classic NK cell effector responses such as ability to secrete pro-inflammatory cytokines and mediate cell cytotoxicity are optimal. Further differentiation into Mature 2 NK cells accompanies a reduction in NK cell effector responses. Loss of DNAM-1 expression at either the Mature 1 or Mature 2 stage of NK cell development is also associated with a loss of classic NK cell effector responses but an increase in ability to produce MIP-1α/β.

The study raises many new and interesting issues about the differentiation and function of NK cells. Foremost, is what is the DNAM-1− subset doing in the naïve tissue and under conditions of immune response. Aligned to this question is where do the DNAM-1^+^ and DNAM-1− NK cell subsets reside geographically in different organs. It would seem likely the DNAM-1^+^ subset would have access to antigen presenting cell (APC) populations given the important role of DNAM-1 in functioning of the immune synapse and the generation of effector cytokines. The DNAM-1− NK cell subset may be positioned to alert other leukocytes through its secretion of MIP-1 species in a non-synapse dependent manner (but cytokine dependent). Presumably these secretion events are post differentiation of the DNAM-1^+^ NK cell into a DNAM-1− NK cells - so in a sense these DNAM-1− NK cells are forming a secondary and/or regulatory response. It will be important to assess the behavior of these NK cell subsets in the presence of various APC populations both ex-vivo and *in vivo*.

The next major question is whether the same differentiation state exists in human NK cell subsets, where the major markers of discriminating NK cells have been CD16^+/−^ and CD56^bright/dim^ subsets of CD3/ TCR negative (also now NKp46^+^) NK cells. DNAM-1 is not expressed in a bimodal fashion on human NK cells, but rather a broadly heterogeneous expression from high to low/negative is observed. These various subsets of NK cells can now be examined to determine whether the same cytokine/chemokine signatures can be found amongst human NK cells subsets that express high or low/no DNAM-1. If these functionally unique human NK cell states can be found then the prospect of translating these findings into clinical benefit will increase. NK cell transfer is increasingly being considered in the treatment of human blood malignancies and may even have merit in protection from or resolution of some virus or other pathogen infections.
